# Systemic Administration of Glibenclamide Fails to Achieve Therapeutic Levels in the Brain and Cerebrospinal Fluid of Rodents

**DOI:** 10.1371/journal.pone.0134476

**Published:** 2015-07-30

**Authors:** Carolina Lahmann, Holger B. Kramer, Frances M. Ashcroft

**Affiliations:** Department of Physiology, Anatomy and Genetics and OXION, University of Oxford, Oxford, United Kingdom; Hungarian Academy of Sciences, HUNGARY

## Abstract

Activating mutations in the Kir6.2 (*KCNJ11*) subunit of the ATP-sensitive potassium channel cause neonatal diabetes (ND). Patients with severe mutations also suffer from neurological complications. Glibenclamide blocks the open K_ATP_ channels and is the treatment of choice for ND. However, although glibenclamide successfully restores normoglycaemia, it has a far more limited effect on the neurological problems. To assess the extent to which glibenclamide crosses the blood-brain barrier (BBB) *in vivo*, we quantified glibenclamide concentrations in plasma, cerebrospinal fluid (CSF), and brain tissue of rats, control mice, and mice expressing a human neonatal diabetes mutation (Kir6.2-V59M) selectively in neurones (nV59M mice). As only small sample volumes can be obtained from rodents, we developed a highly sensitive method of analysis, using liquid chromatography tandem mass spectrometry acquisition with pseudo-selected reaction monitoring, achieving a quantification limit of 10ng/ml (20nM) glibenclamide in a 30μl sample. Glibenclamide was not detectable in the CSF or brain of rats after implantation with subcutaneous glibenclamide pellets, despite high plasma concentrations. Further, one hour after a suprapharmacological glibenclamide dose was administered directly into the lateral ventricle of the brain, the plasma concentration was twice that of the CSF. This suggests the drug is rapidly exported from the CSF. Elacridar, an inhibitor of P-glycoprotein and breast cancer resistance protein (major multidrug resistance transporters at the BBB), did not affect glibenclamide levels in CSF and brain tissue. We also identified a reduced sensitivity to volatile anaesthetics in nV59M mice and showed this was not reversed by systemic delivery of glibenclamide. Our results therefore suggest that little glibenclamide reaches the central nervous system when given systemically, that glibenclamide is rapidly removed across the BBB when given intracranioventricularly, and that any glibenclamide that does enter (and is below our detection limit) is insufficient to influence neuronal function as assessed by anaesthesia sensitivity.

## Introduction

Glibenclamide is a sulphonylurea drug that has been used for the treatment of type 2 diabetes for over sixty years [1]. It acts by inhibiting ATP-sensitive potassium (K_ATP_) channels in pancreatic beta-cells, which stimulates insulin secretion and thereby lowers the blood glucose concentration [2]. The recent discovery that more than 50% of cases of neonatal diabetes are caused by gain-of-function mutations in the K_ATP_ channel has made glibenclamide the treatment of choice for this disease [3,4].

K_ATP_ channels are also expressed in multiple other tissues, including the nervous system, heart, vasculature and skeletal muscle [5]. As a consequence, patients with severe activating K_ATP_ channel mutations present with neurological symptoms in addition to neonatal diabetes (a condition known as DEND syndrome: *d*evelopmental delay, *e*pilepsy and *n*eonatal *d*iabetes) [6–8]. The extent to which neurological function is improved by glibenclamide is unclear. In some patients, limited improvement in motor and cognitive function is observed after initiation of therapy [9–14]. However, for many DEND patients, glibenclamide and other sulphonylureas are ineffective at improving neurological function even when they successfully control the diabetes [9,15–19].

A possible explanation for the failure of glibenclamide to restore neurological function in DEND patients is that the drug does not reach a high enough concentration in the cerebrospinal fluid (CSF) and brain to block the overactivity of mutant K_ATP_ channels. Previous studies using tolbutamide, another sulphonylurea, in an *in vitro* model of the blood-brain barrier (BBB), have shown that the drug is transported across the BBB by a saturable transcellular mechanism [20]. Recent *in situ* brain perfusion studies in mice showed low cerebral accumulation of [^3^H]glibenclamide [21], which was increased 3-fold by inhibition of P-glycoprotein (P-gp) and breast cancer resistance protein (BCRP), suggesting P-gp and BCRP may be involved in efflux of the sulphonylurea from the brain. *In vitro* models of other cellular barriers also support the idea that glibenclamide may be a substrate of these ABC transporters [22,23]. However, no effect of P-gp or BCRP inhibition on [^11^C]glibenclamide was observed in baboons using PET imaging [21].

In this study, we explore the extent to which glibenclamide can accumulate *in vivo* in the brain of rodents when given either subcutaneously or intracranioventricularly. To do so, we developed a method of determining glibenclamide concentrations in the limited sample volumes available. We use a mouse model of DEND syndrome (nV59M mice) to determine if subcutaneous or intracranioventricular administration of glibenclamide can affect neurological function. Our results reveal that despite high plasma levels of glibenclamide, the drug concentration in the CSF remains very low. We also describe an impaired sensitivity to volatile anaesthetics in nV59M mice and show this is unaffected by high plasma levels of glibenclamide. This suggests drug levels are too low to restore this measure of neuronal function fully. Our findings have implications for the management of DEND syndrome.

## Materials and Methods

### Animal care

Work was conducted in accordance with the 1986 UK Animals (Scientific Procedures) Act and University of Oxford ethical guidelines (UK Home Office project licence number: 30/2668) following NC3Rs ARRIVE guidelines (S1 Appendix). Mice (11–14 week old females and males, 25–30g; n = 167 mice) and rats (young adult Lister-hooded males, 200–300g; n = 84 rats) were housed in same-sex littermate groups in a specific-pathogen-free facility in a temperature- and humidity-controlled room on a 12h light-dark cycle (lights on at 7am) with *ad libitum* access to water, food, bedding, and environmental enrichment. Mice were housed in individually ventilated microinsulator cages while rats were housed in open-top cages. Experiments were carried out on mice with selective neural expression of a Kir6.2-V59M mutation (nV59M mice), which were generated in house as previously described [24]. Littermates (ROSA-V59M^+/-^, Nes-Cre^+^, and WT) were used as controls. Genotypes were identified as described earlier [24,25]. Mice were backcrossed to C57Bl/6J for more than 5 generations. All experiments were carried out in the animal facility during the light part of the animals’ light-dark cycle. All animals were drug- and test-naïve at the start of the experiments. All experiments were conducted blinded to the genotype of the mice and any drug treatment. Where possible, half of the animals received experimental treatment (glibenclamide) and half received vehicle. Animals were randomly allocated to either treatment group using computer-generated random numbers (Microsoft Excel).

### Glibenclamide therapy

#### Subcutaneous delivery

Animals were anaesthetized with 2% isoflurane in 100% medical oxygen. The depth of anaesthesia was monitored throughout the procedure by firm pinching of the hindpaws to assess the presence of a withdrawal reflex. Animals were administered buprenorphine (0.05mg/kg subcutaneously; Vetergesic, Reckitt Benckiser Healthcare) and bupivacaine (0.25% at incision site; Marcain, AstraZeneca) pre-operatively. They were then implanted subcutaneously between the scapulae with a 21-day slow-release pellet containing either glibenclamide or vehicle (Innovative Research of America; Mice: 0.25mg, 2.5mg or 25mg pellets; Rats: 25mg or 200mg pellets). Animals were allowed to recover for 7–10 days. Ten rats were implanted with glibenclamide (n = 5 for 25mg pellets; n = 5 for 200mg pellets) and 10 rats with vehicle. For mice, 19 animals were implanted with vehicle and 21 with glibenclamide (n = 5 for 0.25mg pellets; n = 11 for 2.5mg pellets; n = 5 for 25mg pellets).

#### Acute intracranioventricular (ICV) delivery

Rats (n = 10) were anaesthetized with 2–3% isoflurane in 100% medical oxygen and the depth of anaesthesia was monitored throughout the procedure by firm pinching of the hindpaws to assess the presence of a withdrawal reflex. Animals were administered buprenorphine (0.05mg/kg subcutaneously; Vetergesic, Reckitt Benckiser Healthcare) and bupivacaine (0.25% at incision site; Marcain, AstraZeneca) pre-operatively. Using a 10μl Hamilton syringe, 5μl of glibenclamide dissolved in DMSO (25mg/ml; n = 6) or 5μl of vehicle alone (DMSO; n = 4) was injected into the right lateral ventricle of the brain. Animals were sacrificed 1-hour after the injection.

#### Continuous ICV delivery

Animals (rats: n = 10; mice: n = 39) were anaesthetized with 2–3% isoflurane in 100% medical oxygen and implanted with a subcutaneous osmotic mini-pump (Rats: model 2ML4, 2.5μl/h flow rate; Mice: model 2004, 0.25μl/h flow rate; Alzet, Durect Co.) connected to a cannula (Rats: Brain Infusion Kit II; Mice: Brain Infusion Kit III; Alzet, Durect Co.) implanted in the right lateral ventricle (Rats: 0.9mm caudal and 1.3mm lateral from bregma, 4.5mm below the surface of the skull; Mice: 0.1mm caudal and 0.9mm lateral from bregma; 3mm below the skull surface). The pump was filled with either glibenclamide (Rats: 54μg/ml; Mice: 138μg/ml) in artificial cerebrospinal fluid (aCSF) or vehicle (aCSF; 0.1% DMSO). The aCSF contained (in mM): 143 NaCl, 1.2 CaCl_2_, 2.7 KCl, 1 MgCl_2_, 0.26 NaH_2_PO_4_, 1.74 Na_2_HPO_4_, pH 7.4 and 1mg/ml bovine serum albumin. Glibenclamide (Sigma) was made up as a stock solution in 100% DMSO and diluted 100-fold in aCSF (final DMSO concentration 0.1%). Animals were administered buprenorphine (30μg/ml; Vetergesic, Reckitt Benckiser Healthcare) and bupivacaine (0.25%; Marcain, AstraZeneca) pre-operatively. They were allowed to recover for one week before experiments (glibenclamide measurement, neurological function). Cannula placement was confirmed for all animals by standard histological methods (cresyl violet staining). Images were acquired with a Leica light microscope and a Nikon digital camera.

#### Intraperitoneal (IP) delivery of elacridar and glibenclamide

Rats were injected intraperitoneally with 10mg/kg elacridar (Tocris) dissolved in DMSO (n = 11). Controls rats received equal volumes of DMSO (n = 10). One hour later, rats were given a second IP injection of 50mg/kg glibenclamide in DMSO. Four hours after glibenclamide administration, animals were sacrificed.

#### Plasma, CSF and brain sample collection

Animals were killed with an overdose of sodium pentobarbital (150mg/kg; Euthatal, Merial) injected intraperitoneally. Blood was collected from the posterior vena cava into a heparinised syringe and plasma separated by centrifugation (1000 x g, 20min, 4°C). CSF was collected from the cisterna magna with a 0.3ml syringe (BD Micro-Fine+, BD Diabetes). The whole brain was removed, washed with phosphate buffered saline (PBS; Sigma) and homogenised. The homogenate was diluted 1:2 (w/v) with PBS. Samples were collected and stored in 1.5ml polypropelene tubes (Eppendorf) at -20°C.

#### Glibenclamide determination

Blood and CSF samples were analysed by LC-MS/MS on an Ultimate 3000 HPLC system (Dionex, Camberley, UK) coupled via the standard electrospray ionisation (ESI) interface to an Amazon ion trap mass spectrometer (Bruker Daltonics) equipped with a nitrogen generator (Dominick Hunter, LCMS20-1). The HPLC system consisted of a solvent rack and degasser (SRD-3600), a dual gradient pump (DGP-3600M), a flow manager (FLM-3100) and a well plate auto-sampler (WPS-3000T). The mass spectrometer was controlled by TrapControl software version 7.0 and the LC-MS/MS system was controlled by HyStar software version 3.2. Quantification was conducted in Selected Reaction Monitoring (SRM) mode to observe two mass transitions: 494.1→369.0 and 505.1→369.0 for detection of glibenclamide and d_11_-glibenclamide, respectively.

#### Preparation of calibration standards and quality controls

For preparation of the plasma calibration standards a stock solution of glibenclamide (Santa Cruz Biological) was made in methanol (1.0 mg/ml). Blank rat plasma (Lampire Biological Laboratories) was spiked with the stock glibenclamide solution at a concentration of 10μg/ml, and a range of standard concentrations was prepared by individual dilutions at the following concentrations (ng/ml): 4000, 3000, 2000, 1000, 500, 200, 100, 50, 20, 5, 1 and no drug. The same standard concentrations were used for the CSF and brain homogenate calibration curves. Artificial CSF was used to dilute the CSF calibration standards. In the case of the brain calibration curve, brains obtained from drug-naïve rats were homogenised and diluted 2:1 (v/w) with phosphate-buffered saline (PBS; Sigma). This homogenate was used to dilute the calibration standards.

Quality control (QC) samples (n = 5) were prepared from a separate glibenclamide stock solution at the following concentration levels (ng/ml): 3500, 350, and 40. A separate calibration curve was run for each set of experimental samples, and each set of samples was analysed in triplicate LC-MS runs.

Deuterated glibenclamide (d_11_-glibenclamide; Santa Cruz) was chosen as an internal standard (IS) because it offers the advantages of near co-elution and similar ionization properties to the analyte. The IS was spiked at a concentration of 333ng/ml into all calibration standard, QC and study samples.

#### Sample preparation and extraction

A 500μl aliquot of brain homogenate was used for further processing. The internal standard (16μl of 10μg/ml d_11_-glibenclamide) was added to the brain sample and 4% *ortho*-phosphoric acid (85% w/w; Fluka) was added to a final volume of 766μl. Brain samples were first prepared by liquid-liquid extraction. One volume of water-saturated ethyl acetate (Acros Organics) was added to each sample. These were vortexed and centrifuged at 16000 x g for 2min, and the upper solvent phase was then collected for further processing. This procedure was repeated twice. The samples were evaporated in a vacuum centrifuge at 35°C. The remaining residue was taken up in 10% methanol (100μl; Fisher Scientific) by sonication in an ultrasonic bath (10min). In the case of the plasma and CSF samples, the internal standard (1μl of 10μg/ml d_11_-glibenclamide; Santa Cruz) was added to 30μl of the sample and 4% *ortho*-phosphoric acid (85% w/w; Fluka) was added to a final volume of 62μl.

All samples (including calibration standards and quality controls) were processed by reverse phase C18 solid phase extraction (C18-SPE). Acidified aliquots were diluted with 600μl of wash buffer (94.9% water, 5% acetonitrile, Merck; 0.1% formic acid, Fluka) and applied to a pre-equilibrated 96-well reverse phase C18-SPE extraction plate (50mg C18 resin; Microlute Varian) attached to a Vac Master-96 vacuum manifold (Biotage). Wells were pre-equilibrated with 500μl elution buffer (80% acetonitrile, 19.9% water, 0.1% formic acid) followed by 1000μl of wash buffer. Following sample application, the wells were washed twice with 1000μl of wash buffer and eluted with elution buffer (2 x 200μl). The eluates were transferred into silanized 1.5ml Eppendorf tubes and evaporated in a vacuum centrifuge at 35°C. The remaining residue was taken up in 20% methanol (30μl; Fisher Scientific) by sonication in an ultrasonic bath (10min). Samples were centrifuged (16,100 x g at 4°C) for 15min and the supernatant was transferred to glass LC-MS sample vials (Kinesis).

### Isoflurane and halothane sensitivity measurements

nV59M mice (n = 24) and control littermates (n = 22) were individually placed in a closed acrylic chamber (30cmx18cmx17.5cm; Vet-tech Solutions) that had been previously equilibrated with 2% isoflurane (Abbott Laboratories) or 2% halothane (Merial) mixed with 100% medical oxygen. This gas mixture was continuously perfused at a rate of 1 l/min throughout the experiment. The time the mouse stopped moving after entering the chamber was recorded. To determine if this corresponded to the loss of righting reflex (LORR), the chamber was tilted. If the mouse remained with at least three paws in the air for more than 30s, its righting reflex was considered to be lost. The time taken for this to occur was deemed the LORR. The time taken to lose the hindpaw withdrawal reflex (LOWR) was measured as follows: 30s after the mouse had stopped moving, the interdigital pads of one of the hindpaws were firmly pinched for up to 5s. This procedure was repeated every 30s, on alternate paws, until the withdrawal reflex was lost. Any purposeful movement was considered a positive response. This procedure was carried out prior to and a week after implanting the mice with a slow-release 2.5mg glibenclamide or vehicle pellet subcutaneously. Half of the nV59M mice and half of the control littermates were randomly allocated to the glibenclamide treatment group and the other half was allocated to the vehicle treatment group. The procedure was also conducted before, and one week, after implanting mice with a subcutaneous osmotic mini-pump connected to a cannula inserted into the right lateral ventricle of the brain. The mini-pump contained either 138μg/ml glibenclamide or vehicle. Half of the nV59M mice and half of the control littermates were randomly allocated to the glibenclamide treatment group and the other half was allocated to the vehicle treatment group. The experimenter conducting the isoflurane sensitivity assay and surgical procedures was blinded to the genotype and treatment of all mice.

For nV59M mice and control littermates implanted with subcutaneous slow-release pellets, blood glucose was monitored for 5 days before, and up to 7 days after, pellet implantation. The tail was anaesthetized using lidocaine EMLA topical cream (AstraZeneca), and blood obtained via tail vein puncture. Glucose was measured using a Freestyle Lite handheld glucose meter (Abbott).

A 7-day interval between pellet implantation and anaesthesia sensitivity assessment was chosen to allow the animal to recover fully from the operation. The pharmacokinetics of glibenclamide release from the pellets were not measured as the rate of drug delivery from the subcutaneous pellets is stated to be constant by the manufacturer.

### Data analysis

Analysis of mass spectrometry data was performed using Quantanalysis software version 2.0 (Bruker Daltonics) for chromatograms of mass transitions for glibenclamide and d_11_-glibenclamide. A Gaussian smoothing algorithm (smoothing width 1s, 1 cycle) was applied to the chromatograms and automatic peak detection parameters for glibenclamide and d_11_-glibenclamide were 6.6min for retention time and 0.3min for the retention time window. For the calibration curves, peak area ratios of glibenclamide to d_11_-glibenclamide were plotted against the concentrations. A linear regression analysis of the calibration standards was carried out using the least squares method with a 1/y^2^ weighting. Calibration and experimental samples were analysed by triplicate injection on the LC-MS/MS system and the determined value was taken as the arithmetic mean of the three measurements.

Statistical analysis of anaesthesia data from male and female mice (nV59M or control) indicated no significant differences, so the data were pooled. Similarly, analysis of data from the three groups of control mice (e.g. ROSA-V59M^+/-^, Nes-Cre^+^ and WT) indicated no significant differences, so these data were also pooled. When the data distribution permitted, a Student’s t-test was performed. For parametric data with unequal variances, a t-test with Welch’s correction was used. For non-parametric data, a Mann-Whitney test was performed. Data with two independent variables (genotype and treatment or sex and treatment) were analysed using a two-way ANOVA. For multiple comparisons, a Bonferroni multiple comparison post-test was used. *P*<0.05 was considered statistically significant. Statistical analysis was carried out in Graphpad Prism 6. For comparison of plasma glibenclamide concentrations between female and male mice implanted with slow-release 21-day 2.5mg glibenclamide pellets, power calculations were conducted using the MATLAB function sampsizepwr. These demonstrated the sample size was sufficient to conclude gender differences exist.

## Results

Most current methods of measuring glibenclamide are designed for analysis of human plasma, and typically require ~1000μl for accurate determination. This is considerably more volume than can be obtained from a mouse (~500μl; ~30μl from venesection). Thus, we first developed a practical method of determining glibenclamide levels in small volumes of plasma and CSF.

### Glibenclamide measurement

**Fig 1:**
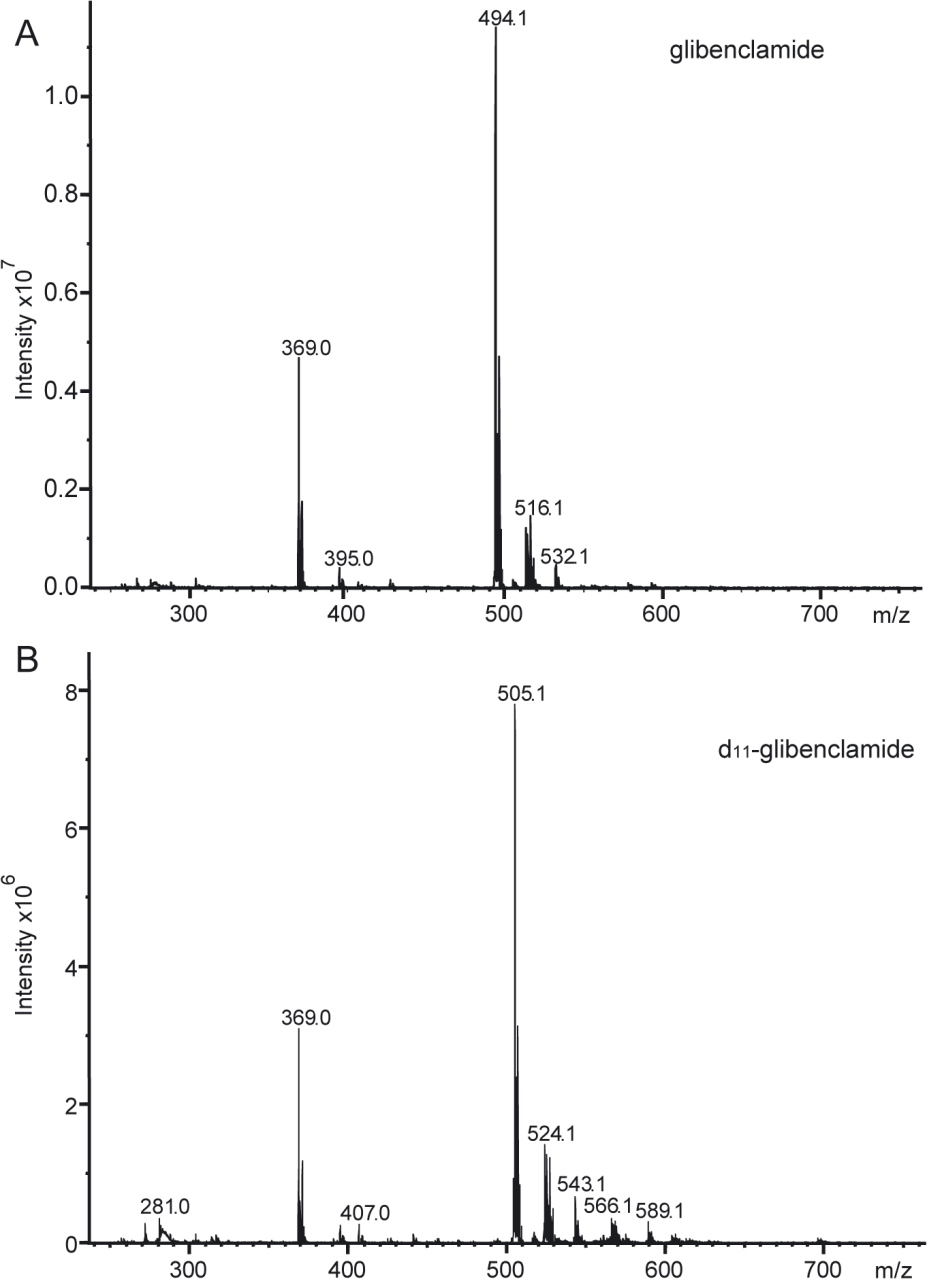
Mass spectra of glibenclamide and d_11_-glibenclamide. (**A,B**) Mass spectra and structural formulae of glibenclamide (**A**) and d_11_-glibenclamide (**B**) in positive ionization mode obtained by direct injection ion trap mass spectrometry. Protonated molecular ions [M+H^+^] m/z (**A**) 494.1, (**B**) 505.1. Fragment ion m/z, (**A,B**) 369.0.

We used a LC-MS/MS mass spectrometry method to measure glibenclamide. Samples were prepared by reverse phase C18 solid phase extraction and d_11_-glibenclamide was used as an internal standard (IS). Selected reaction monitoring (SRM) on an Ion Trap mass spectrometer (pseudo-SRM acquisition [26]), was used to enhance sensitivity. The mass transitions detect fragmentation of protonated precursor ions (494.1 and 505.1) to a common fragment ion (369.0) for glibenclamide and IS (Fig 1). Based on the limit of detection (LOD) we estimate that this method provides a ~100-fold gain in sensitivity relative to detection in full scan MS mode (S1 Fig). No interfering signals were detected at the retention time of the analyte and IS (S1 Fig). The relationship between the spiked glibenclamide concentration and the measured response (ratio of glibenclamide to d_11_-glibenclamide peak area) was linear over the concentration range 10–4000ng/ml (S2 Fig). Calibration curves obtained for three separate analytical runs on different days were linear with a correlation coefficient (r) greater than 0.99 (S2 Fig). The accuracy and precision of the assay were within the ±15% throughout the concentration range 10ng/ml to 4000ng/ml (S1 Table). The performance of the assay was tested with four separately spiked QC samples at three different concentration levels: 3500, 350 and 40 ng/ml (S2 Table). Intra-day assay accuracy (n = 5) and precision were checked by running calibration standards and QC samples on the same day, and were within ±20% on all three days of analysis. Inter-day accuracy (n = 15) and relative standard deviation and assay precision (%CV) were within ±15% (S2 Table). A lower limit of quantification in plasma of 10ng/ml (20nM), as defined by <20% accuracy limit, was achieved reproducibly.

### Glibenclamide concentration in plasma

**Fig 2:**
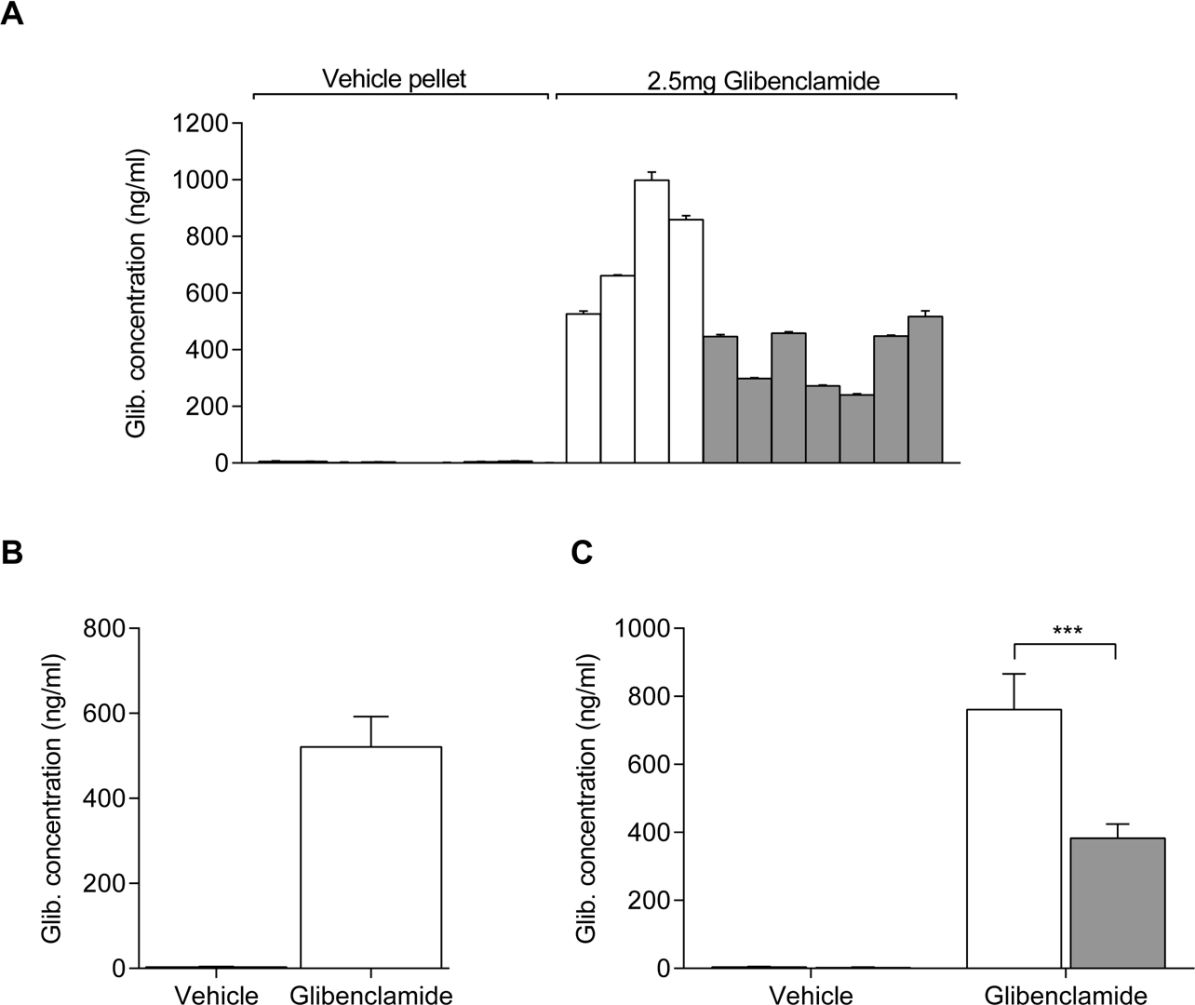
Plasma glibenclamide concentrations. (**A**) Glibenclamide concentration in plasma from individual female (white bars) and male (grey bars) mice implanted with a 21-day slow release 2.5mg glibenclamide pellet or a vehicle pellet. Data are mean±SEM of triplicate measurements. (**B**) Mean±SEM determined glibenclamide concentration in plasma from mice treated with a vehicle (n = 9 animals) or a 21-day slow release 2.5mg glibenclamide (n = 11 animals) pellet. (**C**) Mean±SEM determined glibenclamide concentration in plasma from female (white bars) and male (grey bars) mice implanted with a vehicle (n = 4 and n = 5 animals, respectively) or a 21-day slow release 2.5mg glibenclamide (n = 4 and n = 7 animals, respectively) pellet. ***P<0.0001 [Two-way ANOVA followed by Bonferroni multiple comparison post-test].

Having determined we can quantify glibenclamide accurately at concentrations greater than 10ng/ml in ~30μl samples, we next analysed plasma samples from mice and rats. This revealed the glibenclamide concentration in mouse plasma, 7–10 days after subcutaneous implantation with a 2.5mg/21-day slow-release glibenclamide pellet (dose: ~5mg/kg/day), was between 350 and 2000ng/ml (Fig 2A). No signal was detected in mice implanted with a vehicle pellet (Fig 2B). This drug concentration was chosen as it approximates the maximal oral dose in neonatal diabetes patients treated with glibenclamide (~3mg/kg/day; [27]).

Interestingly, there was a significant difference between male and female mice, with females having approximately twice the mean plasma drug concentration than males (762±105ng/ml versus 383±41ng/ml respectively; *P*<0.0001; Fig 2C). Sex differences have been documented for a number of drugs previously [28] and are frequently explained by differences in pharmacokinetics: in rodents, differential expression of hepatic P450 enzymes that leads to sex-dependent drug metabolism can often be pronounced [29].

**Fig 3:**
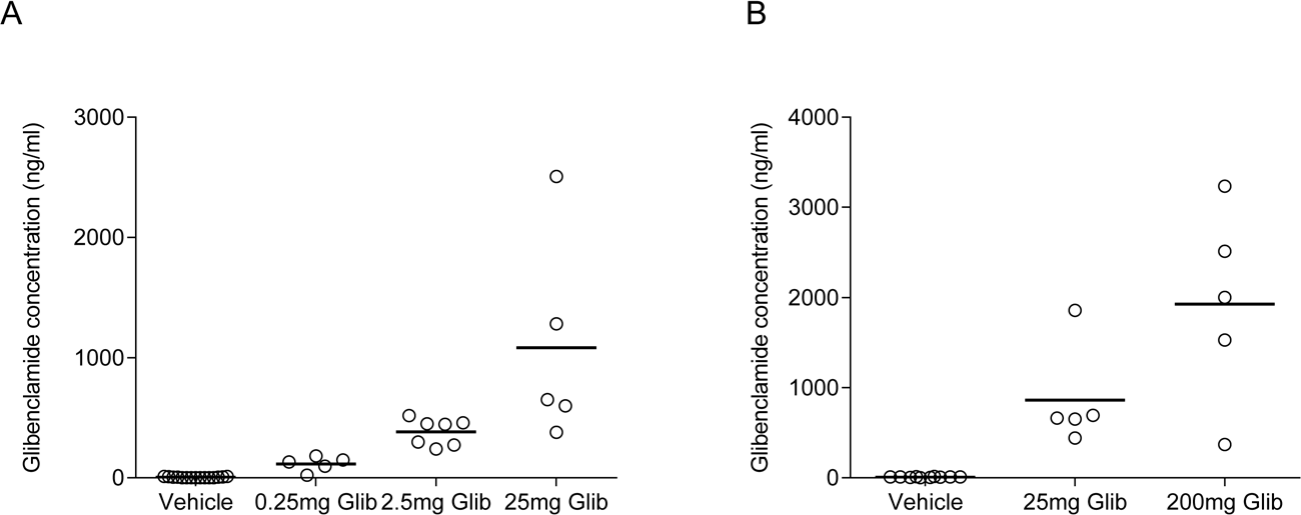
Effect of pellet drug dose on plasma glibenclamide concentration. **(A)** Glibenclamide concentration in plasma from individual male mice implanted with a vehicle (n = 15 animals), a 0.25mg glibenclamide (n = 5 animals), a 2.5mg glibenclamide (n = 7 animals) or a 25mg glibenclamide pellet (n = 5 animals). **(B)** Glibenclamide concentration in plasma from individual male rats implanted with a vehicle (n = 10 animals), a 25mg glibenclamide (n = 5 animals) or a 200mg glibenclamide pellet (n = 5 animals). Data are measurements from individual animals. The mean of each group is represented by the black bar.

We also determined mean plasma glibenclamide concentrations in male mice (Fig 3A) and rats (Fig 3B) implanted with different sizes of slow release pellets. Mice treated with a 0.25mg glibenclamide pellet (dose: ~0.5mg/kg/day) had a mean plasma concentration of 116±27ng/ml (n = 5) while those implanted with a 25mg pellet (dose: ~50mg/kg/day) had a mean concentration of 1084±386ng/ml (n = 5). In the case of the rats, a mean plasma concentration of 863±253ng/ml was obtained for those implanted with a 25mg pellet (dose: ~5mg/kg/day; n = 5), and a mean concentration of 1930±482ng/ml in rats treated with a 200mg glibenclamide pellet (dose: ~50mg/kg/day; n = 5). Thus there is no linear relation between the drug amount in the pellet and the concentration in blood plasma. These results are not unexpected since plasma concentrations are dependent on the rate of release of the drug from the pellet and the rate of clearance from plasma. While the former should ideally scale linearly with the amount of drug in the pellet, the rate of plasma clearance is concentration dependent for compounds that do not follow zero-order elimination kinetics. Glibenclamide elimination kinetics has been described by a first-order rate constant [3].

### Glibenclamide levels in brain and CSF

We next measured glibenclamide levels in the brain and CSF. Rats were used for these experiments as the volume of CSF that can be collected from mice (5–10μl) was below that required for accurate quantification (30μl). Adult male rats were implanted subcutaneously with 200mg glibenclamide slow-release pellets (dose: ~50mg/kg/day) and their plasma, CSF, and brains collected 7–10 days after the surgical procedure. Glibenclamide-implanted rats had a mean plasma glibenclamide concentration of 1.5±0.2μg/ml (n = 11). By contrast, negligible concentrations of glibenclamide were detected in the CSF and brain homogenate samples, all values being below the quantification limit of the method. No glibenclamide was detected in samples from rats implanted with pellets containing the vehicle alone.

**Fig 4:**
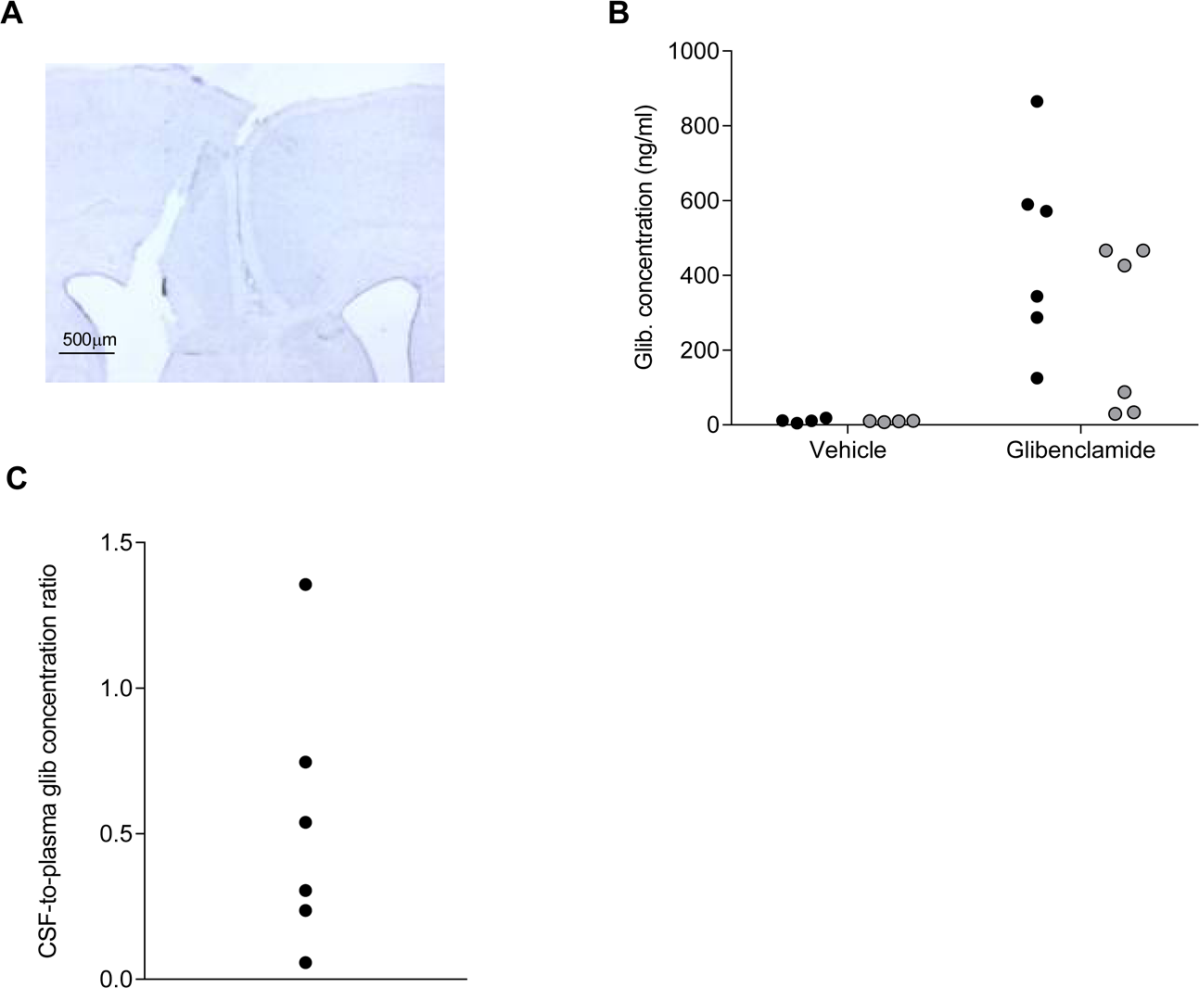
Glibenclamide quantification after intracranioventricular drug delivery. (**A**) Representative photograph of Nissl stained coronal section from a chronically ICV implanted rat (Scale bar: 500μm). (**B**) Glibenclamide concentrations in plasma (black circles) and CSF (grey circles) of rats given vehicle (n = 4) or glibenclamide (n = 6) by acute ICV injection. (**C**) CSF-to-plasma glibenclamide concentration ratio of rats given glibenclamide by acute ICV injection. All data are measurements from individual rats.

We also examined the effect of intracranioventricular (ICV) delivery of glibenclamide using an implanted osmotic mini-pump filled with 54μg/ml glibenclamide. Appropriate placement of the cannulae was confirmed histologically (Fig 4A). Surprisingly, no drug was detected in either the plasma or CSF of glibenclamide-treated rats, all values being below the quantification threshold. Glibenclamide is notorious for binding to non-polar surfaces such as polythene tubing, as well as to plasma proteins [3] so to reduce drug binding to the mini-pump, tubing and cannula we included 1mg/ml of bovine serum albumin in the infusate in all experiments. In separate experiments, we measured the glibenclamide concentration in the solution extruded from the infusion apparatus (mini-pump, tubing and cannula) over a 7-day period. This gave values of 16 and 12.3μg/ml. Thus the glibenclamide concentration infused into the brain will be less than that with which the mini-pump was filled but is still far in excess of that required to fully block the K_ATP_ channel [30]. It is also ~1000-fold higher than the detection threshold of our assay (10ng/ml). Therefore, our inability to detect glibenclamide in the plasma and CSF of ICV-implanted rats is unlikely to have been caused by a lack of glibenclamide in the pump effluate.

One possible explanation for the absence of measureable glibenclamide is that the drug is exported from the CSF as soon as it enters. Similarly, the lack of glibenclamide in the plasma might simply reflect the fact that any drug that crosses the BBB and enters the systemic circulation is diluted to levels below the assay quantification threshold by the much larger blood volume. This hypothesis was tested by acutely injecting a high concentration of glibenclamide (5μl of a 25mg/ml solution) into the right lateral ventricle of rats. With this protocol, glibenclamide was detected in both the CSF and the plasma one hour after drug administration (Fig 4B). Interestingly, for most animals, the plasma concentration was almost twice that of the CSF, consistent with the idea that the drug is rapidly removed from the CSF (Fig 4C).

**Fig 5:**
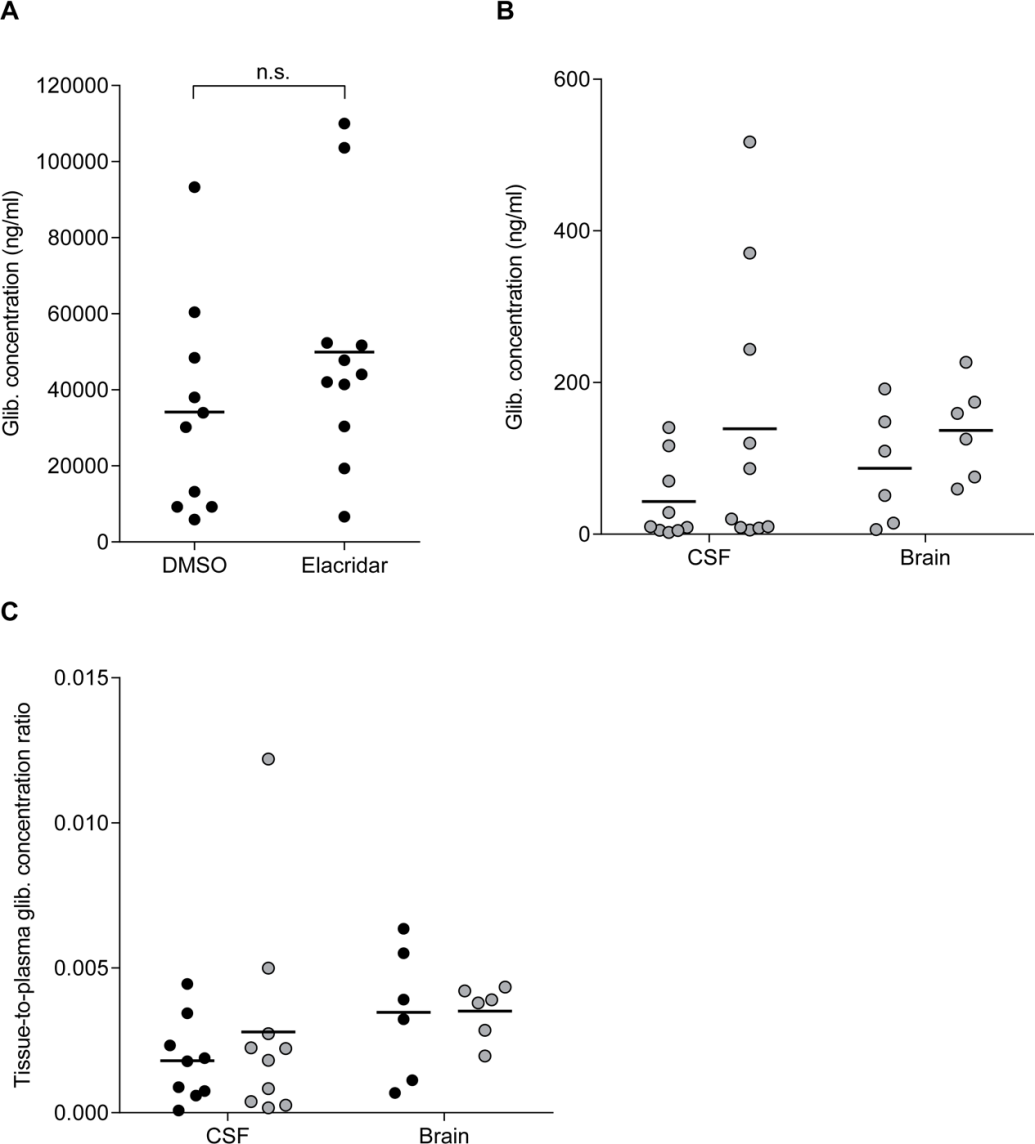
Effect of P-glycoprotein inhibition on glibenclamide concentrations. (**A**) Individual glibenclamide concentrations in plasma of rats injected intraperitoneally with 50mg/kg glibenclamide in the presence of elacridar (n = 11; grey bars) or vehicle (n = 10; black bars). (**B**) Individual glibenclamide concentrations measured in the CSF and brain homogenate of the rats injected with glibenclamide in the presence of elacridar (grey circles; n = 10 for CSF, n = 6 for brain) or vehicle (black circles; n = 9 for CSF, n = 6 for brain). (**C**) CSF-to-plasma and brain-to-plasma glibenclamide concentration ratio of rats injected IP with glibenclamide in the presence of elacridar (grey circles) or vehicle (black circles). Data are measurements from individual rats. The mean of each group is represented by the black bar. n.s. not statistically significant [Two-tailed unpaired Student’s t-test].

In vitro studies suggest that glibenclamide is transported across the BBB by P-glycoprotein (P-gp) [23,31]. To test if this is the case *in vivo*, rats were given 50mg/kg glibenclamide by intraperitoneal (IP) injection in the presence or absence of elacridar, a P-gp and BCRP inhibitor [32–34]. In the absence of elacridar, the plasma glibenclamide concentration was 34μg/ml following IP glibenclamide injection (Fig 5A). Interestingly, glibenclamide was also detected in the CSF and brain homogenate (Fig 5B), reaching levels of 31±21ng/ml (n = 9) and 85±32ng/ml (n = 6), respectively. Thus when administered systemically at a suprapharmacological concentration, the drug can reach the CSF and brain tissue, although at levels substantially lower than those in plasma (Fig 5C). Elacridar did not affect plasma glibenclamide concentrations as drug levels were not significantly different in rats treated with glibenclamide in the presence or absence of elacridar (Fig 5A). Thus elacridar is unlikely to influence glibenclamide clearance/metabolism. There was no significant difference in glibenclamide levels in CSF and brain homogenate samples from elacridar- and vehicle-treated rats (Fig 5B).

### Effects of glibenclamide on neurological function

**Fig 6:**
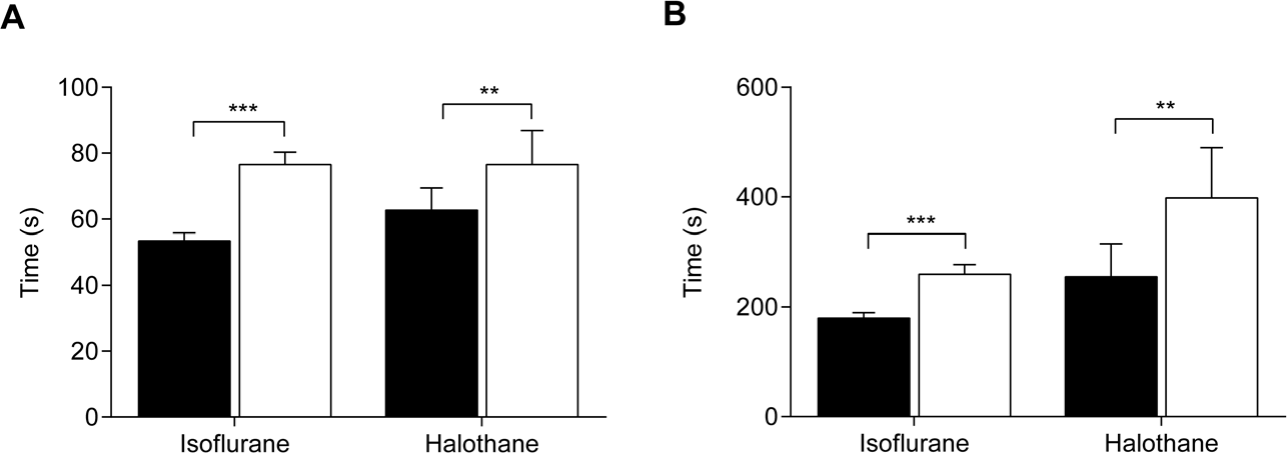
Sensitivity to isoflurane and halothane anaesthesia of nV59M mice. Time taken for loss of righting (**A**) and withdrawal (**B**) reflexes in response to 2% isoflurane or 2% halothane in 11–14 week-old nV59M mice (white bars; isoflurane: n = 41; halothane: n = 9) and control littermates (black bars; isoflurane: Nes-Cre^+^ n = 18, WT n = 15, ROSA-V59M^+/-^ n = 27; halothane: Nes-Cre^+^ n = 5; WT n = 2; ROSA-V59M^+/-^ n = 7). All data are mean ±SEM. ** *P*<0.001; *** *P*<0.0001 [Isoflurane: Mann-Whitney test; halothane: two-tailed unpaired t-test with Welch’s correction].

Our results show that glibenclamide levels in the brain and CSF are very low despite very high plasma levels. Nevertheless, it is possible that concentrations below the LOD of our assay, if present, might affect brain function. To explore if this might be the case, we compared the behaviour of wild-type mice and nV59M mice, a mouse model of DEND syndrome carrying the Kir6.2-V59M mutation [24], in the presence and absence glibenclamide. As shown in Fig 6, nV59M mice show an impaired sensitivity to volatile general anaesthetics: when exposed to isoflurane or halothane anaesthesia, they take ~30% longer than control littermates to lose their righting (LORR; an indication of sedation; Fig 6A) and withdrawal reflexes (LOWR; a sign of pain awareness; Fig 6B). As sensitivity to general anaesthesia is a robust, rapid and non-invasive assay, we used this phenotype to assess the effect of systemically administered glibenclamide therapy on neurological function. The response to isoflurane anaesthesia was examined a week before and a week after implanting nV59M and control mice with either a vehicle or a slow-release 2.5mg glibenclamide pellet (dose: ~5mg/kg/day).

**Fig 7:**
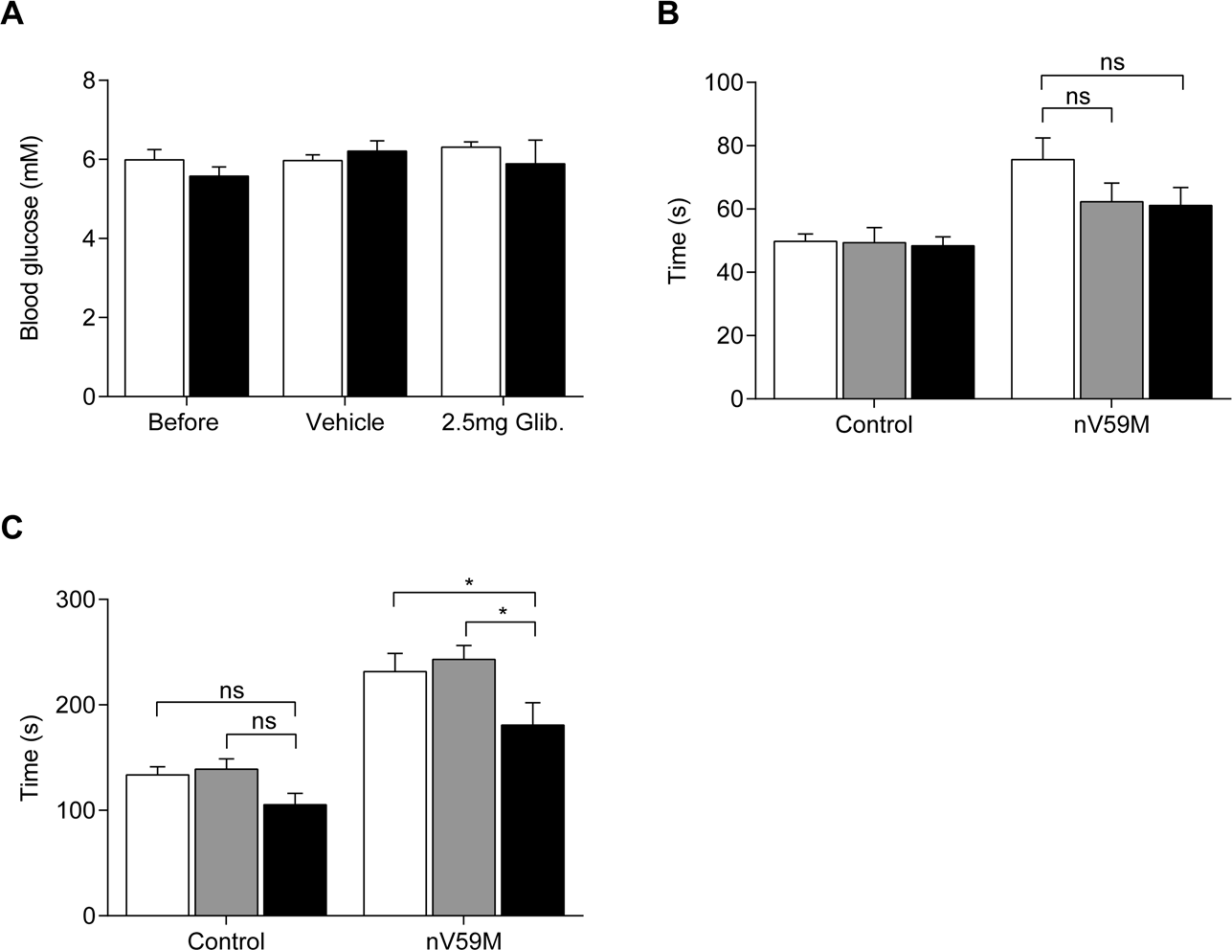
Effect of subcutaneous glibenclamide therapy on blood glucose and isoflurane anaesthesia. (**A**) Free-fed blood glucose concentration of nV59M mice (black bars; n = 24) and control littermates (white bars; WT, n = 7; Nes-Cre^+^, n = 5; ROSA-V59M^+/-^, n = 10) before and after implanting with a vehicle or 2.5mg glibenclamide slow-release subcutaneous pellet. The mean blood glucose of each mouse was averaged over a period of 5 days before and up to 7 days after pellet implantation. (**B,C**) Time taken for loss of righting (**B**) and withdrawal (**C**) reflexes in response to 2% isoflurane anaesthesia before (white bars) and one week after implanting nV59M mice (n = 24) and control littermates (WT, n = 7; Nes-Cre^+^, n = 5; ROSA-V59M^+/-^, n = 10) with either a vehicle (grey bars) or 2.5mg glibenclamide (black bars) subcutaneous pellet. Half of the mice from each group were implanted with a vehicle pellet and the other half with glibenclamide. All data are mean±SEM. * P<0.05; n.s. not statistically significant. [Two-way ANOVA (genotype x treatment) followed by Bonferroni multiple comparison post-test].

Glibenclamide did not significantly affect the blood glucose concentration of either nV59M or control mice (Fig 7A), as previously reported [35]. Neither vehicle nor glibenclamide altered the LORR of control animals (Fig 7B). In nV59M mice, both vehicle and glibenclamide decreased the LORR but this was not statistically significant (Fig 7B). Thus, subcutaneous glibenclamide therapy fails to restore the impaired LORR of nV59M mice. In contrast, glibenclamide treatment partially restored the impaired LOWR of nV59M mice (*P* = 0.0323; two-way ANOVA; Fig 7C) but not control mice. Vehicle treatment was ineffective on the LOWR of both control and mutant mice.

**Fig 8:**
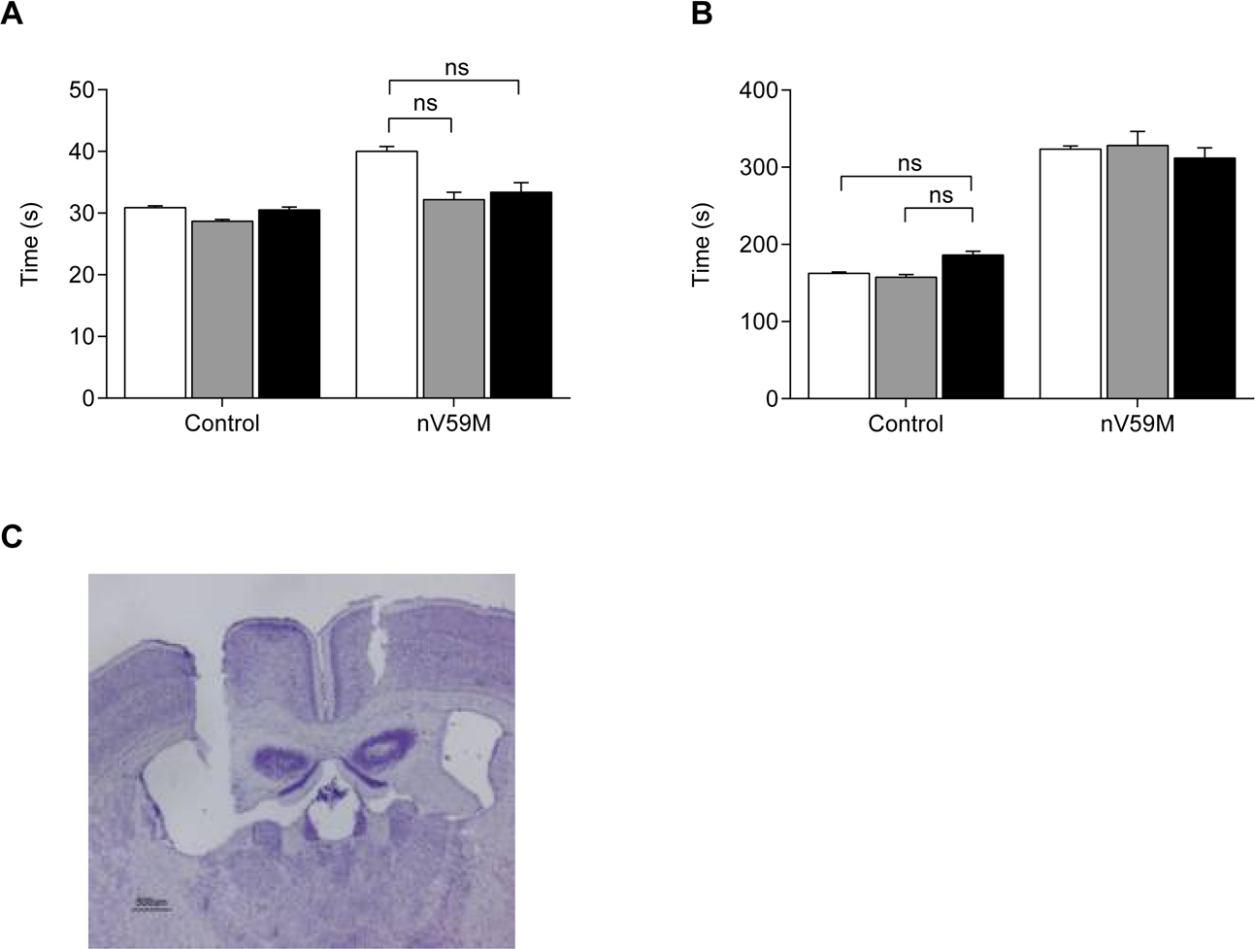
Effect of intracranioventricular glibenclamide on the sensitivity to isoflurane. (**A**) Time taken for the LORR before (white bars) and one week after ICV infusion of vehicle (grey bars) or glibenclamide (138μg/ml, black bars) in nV59M (n = 12) and control (n = 27) mice. (**B**) Time taken for the LOWR before (white bars) and one week after ICV infusion of vehicle (grey bars) or glibenclamide (138μg/ml, black bars) in nV59M (n = 12) and control (n = 27) mice. (**C**) Representative Nissl-stained coronal section of mouse brain showing the site of the ICV cannula. (Scale bar: 500μm). Data are mean±SEM. n.s. not statistically significant [Two-way ANOVA (genotype x treatment) with *post-hoc* Bonferroni test].

We also examined the effect of intracranioventricular infusion of glibenclamide on the sensitivity to isoflurane anaesthesia. ICV delivery of glibenclamide was ineffective at restoring either the impaired LORR (Fig 8A) or LOWR (Fig 8B) of nV59M mice, despite appropriate placement of the cannulae (Fig 8C). Neither ICV glibenclamide nor vehicle altered the response of control mice to isoflurane. This may be because the slow infusion rate (dictated by the method) was insufficient to compensate for the rapid rate of drug efflux from the brain. The lack of a glibenclamide effect on the LOWR of nV59M mice when given by ICV infusion (Fig 8B), but partial restoration of the response when given subcutaneously (Fig 7C) is consistent with the effect of drug being mediated on neuronal tissues outside BBB. These results are in line with our glibenclamide measurements following ICV drug delivery in rats, which showed levels below the LOD of our method in both the CSF and plasma despite high concentrations being released by the osmotic mini-pump.

## Discussion

Our results show that glibenclamide does not readily reach the CSF or brain tissue of rats when administered subcutaneously, despite very high concentrations (19μg/ml) being found in the plasma. To bypass the BBB, glibenclamide (54μg/ml) was delivered directly into the right lateral ventricle. However, no drug was detected in the plasma, CSF or brain tissue with this protocol, perhaps because the drug is exported from the CSF and any drug that enters the systemic circulation is diluted to levels below the assay quantification threshold by the much larger blood volume as well as by distribution to peripheral tissues. Intraperitoneal injection of a suprapharmacological glibenclamide concentration as a single bolus led to measurable levels in the CSF (31ng/ml), but this was still ~1000-fold lower than that found in the plasma (34 μg/ml). In summary, our results suggest that despite the fact it is a highly lipophilic drug, glibenclamide does not reach significant concentrations in the brain unless the plasma concentration is extremely high. Glibenclamide is well known to bind plasma proteins—~98% of the drug in plasma is bound [3]. This impairs entry of glibenclamide into the brain as plasma proteins are unable to cross the BBB. It is thus likely that only under conditions where the unbound drug concentration is very high (i.e at supra-therapeutic doses) does subcutaneously administered glibenclamide reach measurable concentrations in the brain. Glibenclamide also appears to be rapidly removed from the brain when injected directly into the lateral ventricle. Thus there appears to be a highly efficient efflux system for glibenclamide in the brain. Previous studies have also reported that although sulphonylureas can cross the BBB, they are rapidly extruded [20,21].

One possible candidate for mediating glibenclamide transport across the BBB is P-gp. Earlier *in vitro* experiments have shown that glibenclamide is able to inhibit P-gp and may be also a substrate for this transporter [23,31]. Administration of elacridar, a P-gp and BCRP inhibitor, prior to treatment did not affect the plasma glibenclamide concentrations produced by intraperitoneal injection of the drug. This suggests that transporters other than (or additional to) P-gp and BCRP are involved in the rapid efflux of glibenclamide across the BBB in mice. This is unsurprising given the well-established redundancy in xenobiotic export transporters at the BBB [36–38]. Similar conclusions were recently reached using ^11^C-radiolabelled-glibenclamide positron emission topography in baboons [21].

### Effects of glibenclamide on the LORR and LOWR

In nV59M mice, systemically administered glibenclamide had no significant effect on the LORR, but partially restored the altered LOWR. These data suggest that glibenclamide can access at least some of the neuronal pathways involved in the LOWR, but not those involved in the LORR. The lack of effect of the drug on the LORR could be due to an inability of glibenclamide to reach therapeutic levels in the brain whereas the partial restoration of the LOWR argues that it may involve pathways outside the BBB, such as primary afferent neurones [39].

Administration of glibenclamide via into the lateral ventricle of nV59M and control littermates via an osmotic mini-pump had no effect on anaesthesia sensitivity. The most likely reason for this is that glibenclamide did not reach a high enough level in the brain, as we were unable to measure any quantifiable drug concentration when glibenclamide was administered this way. Unfortunately, we could not increase the drug concentration in the mini-pump solution because it was not soluble at higher concentrations. Thus, we could not determine whether the drug might have been effective at higher doses. Bolus administration of a suprapharmacological dose of glibenclamide (either by acute ICV injection or intraperitoneal injection) led to detectable levels of the drug in the CSF and brain, but this was too transient and too short to enable the animal to recover and be tested for anaesthesia sensitivity. Consequently, we cannot distinguish if a lack of a change in anaesthesia sensitivity is due to too low a drug dose, or because of the absence of K_ATP_ channel activity during development produced irreversible changes in neuronal function.

### Implications for therapy

While it is difficult to directly compare our results and data from patients treated with sulphonylureas, because glibenclamide was administered via different routes and there are pharmacokinetic differences between species, our data show that if the plasma concentration is high enough, glibenclamide may reach the brain and CSF. However, this is only achieved with plasma glibenclamide concentrations at which the drug also blocks other types of ion channels [40], and thus it may have off-target effects in peripheral tissues. It therefore seems unlikely that such high drug doses would be tolerated in patients.

The subcutaneous glibenclamide concentration we administered in rats was ~17 times the maximum oral dose taken by patients with neonatal diabetes (3mg/kg/day in two doses; [27]). Plasma levels in ND patients are also 3–30-fold lower [41]. Thus it seems likely that glibenclamide levels in the brain will be very low (<10ng/ml) in patients with neonatal diabetes. Although 10ng/ml (~20nM) glibenclamide blocks wild-type K_ATP_ channels by ~80%, it is much less effective on mutant K_ATP_ channels [30]. Furthermore, it is the absolute magnitude of the K_ATP_ current that matters functionally–this is difficult to quantify as it will also depend on channel block by intracellularly generated ATP (which will be less for mutant channels). Thus it is not a simple matter to predict K_ATP_ current magnitude *in vivo*. Nevertheless, our data show that even when plasma glibenclamide levels are very high, no effect of the drug is observed on anaesthesia sensitivity. This may be important if, like the mouse model, DEND patients experience reduced sensitivity to inhalation anaesthesia as they may be at risk of anaesthesia awareness. It also seems possible that our data will translate to other types of neurological symptoms.

Our results thus raise the question of whether the drug concentration in the CSF and brain of DEND patients treated with oral glibenclamide is sufficient to block K_ATP_ channels enough to affect neuronal electrical activity. In fact, increased blood flow, an indicator of neuronal activity, was observed only in the cerebellum following high dose glibenclamide therapy [42], although K_ATP_ channels are widely expressed throughout the brain [43,44]. This suggests that electrical activity in the cerebellum may be more sensitive to tiny changes in K_ATP_ channel activity than other brain areas.

## Supporting Information

S1 Appendix NC3Rs ARRIVE Guidelines Checklist for reporting *in vivo* animal experiments. (PDF)

S1 Fig Serial dilution of glibenclamide in blank mouse plasma analysed by pseudoSRM ion trap mass spectrometry. LC-MS chromatograms displaying the intensity of transition m/z 494.1→369.0 (6.6min retention time) for spiked plasma samples with the following concentrations: 7.8, 15.6, 31.3, 62.5, 125, 250, 500, 1000 and 2000ng/ml.
(TIF)

S2 Fig Reproducibility and linearity of calibration with spiked plasma standards. Calibration curves obtained from triplicate analysis of spiked plasma standards by LC-MS (SRM acquisition) immediately prior to injection of unknown samples. Replicates for each set of replicates and the corresponding R^2^ values are displayed in dark blue, green and red.
(TIF)

S3 Fig Different plasma extraction methods have strong influence on levels of phospholipid contamination and matrix effects. Analysis of plasma extracts by nanoflow LC-MS showing extracted ion chromatograms of m/z 520.4 phospholipid contaminant (cyan) and m/z 494.1 glibenclamide (magenta).(**A**,**C**) protein precipitation and filtration (note different scales in **A** and **C**). (**B**,**D**) reverse phase C18-SPE (note different scales in **B** and **D**).
(TIF)

S1 TableAccuracy and precision of plasma calibration standard curves.Calibration curves obtained from triplicate analysis of spiked plasma standards by LCMS (SRM acquisition) on three separate days. Linear regression was carried out by the least squares method with a 1/y^2^ weighting. Precision was expressed as percentage coefficient of variation (%CV) and was calculated as $\frac{standard\;deviation}{mean\;concentration}\times 100$.(DOCX)

S2 TableIntra-day and inter-day accuracy and precision of assay determined with QC samples at three concentration levels.Mean accuracy, relative standard deviation (SD) and coefficient of variation (CV) for intra-day (n = 5) and inter-day measurements (n = 15). Accuracy is defined as the percentage of the theoretical concentration. Relative SD is defined as $\frac{standard\;deviation}{theoretical\;concentration}\times 100$. Precision is expressed as the percentage coefficient of variation (%CV) and calculated as $\frac{standard\;deviation}{mean\;concentration}\times 100$.(DOCX)

## References

[1] Henquin JC. The fiftieth anniversary of hypoglycaemic sulphonamides. How did the mother compound work? Diabetologia. 1992;35: 907–912. 1451945 10.1007/BF00401417

[2] Gribble FM, Reimann F. Sulphonylurea action revisited: the post-cloning era Diabetologia. Springer-Verlag; 2003;46: 875–891. 10.1007/s00125-003-1143-3 12819907

[3] Pearson JG. Pharmacokinetics of glyburide. Am J Med. 1985;79: 67–71. 10.1016/s0002-9343(85)80010-3 3931464

[4] Rafiq M, Flanagan SE, Patch A-M, Shields BM, Ellard S, Hattersley AT, et al Effective treatment with oral sulfonylureas in patients with diabetes due to sulfonylurea receptor 1 (SUR1) mutations. Diabetes Care. American Diabetes Association; 2008;31: 204–209. 10.2337/dc07-1785 18025408 PMC7611807

[5] Seino S, Miki T. Physiological and pathophysiological roles of ATP-sensitive K+ channels. Prog Biophys Mol Biol. 2003;81: 133–176. 12565699 10.1016/s0079-6107(02)00053-6

[6] Gloyn AL, Pearson ER, Antcliff JF, Proks P, Bruining GJ, Slingerland AS, et al Activating mutations in the gene encoding the ATP-sensitive potassium-channel subunit Kir6.2 and permanent neonatal diabetes. N Engl J Med. 2004;350: 1838–1849. 10.1056/NEJMoa032922 15115830

[7] Hattersley AT, Ashcroft FM. Activating mutations in Kir6.2 and neonatal diabetes: new clinical syndromes, new scientific insights, and new therapy. Diabetes. 2005;54: 2503–2513. 16123337 10.2337/diabetes.54.9.2503

[8] Edghill EL, Flanagan SE, Ellard S. Permanent neonatal diabetes due to activating mutations in ABCC8 and KCNJ11. Rev Endocr Metab Disord. Springer US; 2010;11: 193–198. 10.1007/s11154-010-9149-x 20922570

[9] Slingerland AS, Nuboer R, Hadders-Algra M, Hattersley AT, Bruining GJ. Improved motor development and good long-term glycaemic control with sulfonylurea treatment in a patient with the syndrome of intermediate developmental delay, early-onset generalised epilepsy and neonatal diabetes associated with the V59M mutation in the KCNJ11 gene. Diabetologia. Springer-Verlag; 2006;49: 2559–2563. 10.1007/s00125-006-0407-0 17047922

[10] Mlynarski W, Tarasov AI, Gach A, Girard CA, Pietrzak I, Zubcevic L, et al Sulfonylurea improves CNS function in a case of intermediate DEND syndrome caused by a mutation in KCNJ11. Nat Clin Pract Neurol. Nature Publishing Group; 2007;3: 640–645. 10.1038/ncpneuro0640 17982434

[11] Shimomura K, Hörster F, de Wet H, Flanagan SE, Ellard S, Hattersley AT, et al A novel mutation causing DEND syndrome: a treatable channelopathy of pancreas and brain. Neurology. Lippincott Williams & Wilkins; 2007;69: 1342–1349. 10.1212/01.wnl.0000268488.51776.53 17652641

[12] Koster JC, Kurata HT, Enkvetchakul D, Nichols CG. DEND mutation in Kir6.2 (KCNJ11) reveals a flexible N-terminal region critical for ATP-sensing of the KATP channel. Biophys J. Elsevier; 2008;95: 4689–4697. 10.1529/biophysj.108.138685 18708460 PMC2576385

[13] Slingerland AS, Hurkx W, Noordam K, Flanagan SE, Jukema JW, Meiners LC, et al Sulphonylurea therapy improves cognition in a patient with the V59M KCNJ11 mutation. Diabet Med. Blackwell Publishing Ltd; 2008;25: 277–281. 10.1111/j.1464-5491.2007.02373.x 18307455

[14] Battaglia D, Lin Y-W, Brogna C, Crinò A, Grasso V, Mozzi AF, et al Glyburide ameliorates motor coordination and glucose homeostasis in a child with diabetes associated with the KCNJ11/S225T, del226-232 mutation. Pediatr Diabetes. 2012;13: 656–660. 10.1111/j.1399-5448.2012.00874.x 22694282 PMC3747824

[15] Masia R, Koster JC, Tumini S, Chiarelli F, Colombo C, Nichols CG, et al An ATP-binding mutation (G334D) in KCNJ11 is associated with a sulfonylurea-insensitive form of developmental delay, epilepsy, and neonatal diabetes. Diabetes. American Diabetes Association; 2007;56: 328–336. 10.2337/db06-1275 17259376

[16] Gurgel LC, Crispim F, Noffs MHS, Belzunces E, Rahal MA, Moisés RS. Sulfonylrea treatment in permanent neonatal diabetes due to G53D mutation in the KCNJ11 gene: improvement in glycemic control and neurological function. Diabetes Care. American Diabetes Association; 2007;30: e108–e108. 10.2337/dc07-1196 17965292

[17] Manna Della T, Battistim C, Radonsky V, Savoldelli RD, Damiani D, Kok F, et al Glibenclamide unresponsiveness in a Brazilian child with permanent neonatal diabetes mellitus and DEND syndrome due to a C166Y mutation in KCNJ11 (Kir6.2) gene. Arq Bras Endocrinol Metabol. 2008;52: 1350–1355. 19169493 10.1590/s0004-27302008000800024

[18] Zwaveling-Soonawala N, Hagebeuk EE, Slingerland AS, Ris-Stalpers C, Vulsma T, van Trotsenburg AS. Successful transfer to sulfonylurea therapy in an infant with developmental delay, epilepsy and neonatal diabetes (DEND) syndrome and a novel ABCC8 gene mutation. Diabetologia. Springer-Verlag; 2011;54: 469–471. 10.1007/s00125-010-1981-8 21109997 PMC3017308

[19] Itoh S, Matsuoka H, Yasuda Y, Miyake N, Suzuki K, Yorifuji T, et al DEND syndrome due to V59A mutation in KCNJ11 gene: unresponsive to sulfonylureas. J Pediatr Endocrinol Metab. 2013;26: 143–146. 10.1515/jpem-2012-0236 23382304

[20] Takanaga H, Murakami H, Koyabu N, Matsuo H, Naito M, Tsuruo T, et al Efflux transport of tolbutamide across the blood-brain barrier. J Pharm Pharmacol. 1998;50: 1027–1033. 9811164 10.1111/j.2042-7158.1998.tb06918.x

[21] Tournier N, Saba W, Cisternino S, Peyronneau M-A, Damont A, Goutal S, et al Effects of selected OATP and/or ABC transporter inhibitors on the brain and whole-body distribution of glyburide. AAPS J. Springer US; 2013;15: 1082–1090. 10.1208/s12248-013-9514-2 23907487 PMC3787228

[22] Gedeon C, Behravan J, Koren G, Piquette-Miller M. Transport of glyburide by placental ABC transporters: implications in fetal drug exposure. Placenta. 2006;27: 1096–1102. 10.1016/j.placenta.2005.11.012 16460798

[23] Bessadok A, Garcia E, Jacquet H, Martin S, Garrigues A, Loiseau N, et al Recognition of sulfonylurea receptor (ABCC8/9) ligands by the multidrug resistance transporter P-glycoprotein (ABCB1): functional similarities based on common structural features between two multispecific ABC proteins. J Biol Chem. American Society for Biochemistry and Molecular Biology; 2011;286: 3552–3569. 10.1074/jbc.M110.155200 21098040 PMC3030360

[24] Clark RH, McTaggart JS, Webster R, Mannikko R, Iberl M, Sim XL, et al Muscle dysfunction caused by a KATP channel mutation in neonatal diabetes is neuronal in origin. Science. American Association for the Advancement of Science; 2010;329: 458–461. 10.1126/science.1186146 20595581 PMC5890903

[25] Girard CA, Wunderlich FT, Shimomura K, Collins S, Kaizik S, Proks P, et al Expression of an activating mutation in the gene encoding the KATP channel subunit Kir6.2 in mouse pancreatic beta cells recapitulates neonatal diabetes. J Clin Invest. American Society for Clinical Investigation; 2009;119: 80–90. 10.1172/JCI35772 19065048 PMC2613450

[26] Hopfgartner G, Bourgogne E. Quantitative high-throughput analysis of drugs in biological matrices by mass spectrometry. Mass Spectrom Rev. Wiley Subscription Services, Inc., A Wiley Company; 2003;22: 195–214. 10.1002/mas.10050 12838545

[27] Pearson ER, Flechtner I, Njølstad PR, Malecki MT, Flanagan SE, Larkin B, et al Switching from insulin to oral sulfonylureas in patients with diabetes due to Kir6.2 mutations. N Engl J Med. 2006;355: 467–477. 10.1056/NEJMoa061759 16885550

[28] Anderson GD. Sex and racial differences in pharmacological response: where is the evidence? Pharmacogenetics, pharmacokinetics, and pharmacodynamics. J Womens Health (Larchmt). Mary Ann Liebert, Inc. 2 Madison Avenue Larchmont, NY 10538 USA; 2005;14: 19–29. 10.1089/jwh.2005.14.19 15692274

[29] Waxman DJ, Holloway MG. Sex differences in the expression of hepatic drug metabolizing enzymes. Mol Pharmacol. American Society for Pharmacology and Experimental Therapeutics; 2009;76: 215–228. 10.1124/mol.109.056705 19483103 PMC2713118

[30] Proks P, de Wet H, Ashcroft FM. Molecular mechanism of sulphonylurea block of K(ATP) channels carrying mutations that impair ATP inhibition and cause neonatal diabetes. Diabetes. American Diabetes Association; 2013;62: 3909–3919. 10.2337/db13-0531 23835339 PMC3806600

[31] Golstein PE, Boom A, van Geffel J, Jacobs P, Masereel B, Beauwens R. P-glycoprotein inhibition by glibenclamide and related compounds. Pflugers Arch. 1999;437: 652–660. 10087141 10.1007/s004240050829

[32] Allen JD, Brinkhuis RF, Wijnholds J, Schinkel AH. The mouse Bcrp1/Mxr/Abcp gene: amplification and overexpression in cell lines selected for resistance to topotecan, mitoxantrone, or doxorubicin. Cancer Res. 1999;59: 4237–4241. 10485464

[33] Ward KW, Azzarano LM. Preclinical pharmacokinetic properties of the P-glycoprotein inhibitor GF120918A (HCl salt of GF120918, 9,10-dihydro-5-methoxy-9-oxo-N-[4-[2-(1,2,3,4-tetrahydro-6,7-dimethoxy-2-isoquinolinyl)ethyl]phenyl]-4-acridine-carboxamide) in the mouse, rat, dog, and monkey. J Pharmacol Exp Ther. American Society for Pharmacology and Experimental Therapeutics; 2004;310: 703–709. 10.1124/jpet.104.068288 15056727

[34] Karibe T, Hagihara-Nakagomi R, Abe K, Imaoka T, Mikkaichi T, Yasuda S, et al Evaluation of the usefulness of breast cancer resistance protein (BCRP) knockout mice and BCRP inhibitor-treated monkeys to estimate the clinical impact of BCRP modulation on the pharmacokinetics of BCRP substrates. Pharm Res. Springer US; 2015;32: 1634–1647. 10.1007/s11095-014-1563-4 25380981

[35] Remedi MS, Nichols CG. Chronic antidiabetic sulfonylureas in vivo: reversible effects on mouse pancreatic beta-cells. Groop L, editor. PLoS Med. Public Library of Science; 2008;5: e206 10.1371/journal.pmed.0050206 18959471 PMC2573909

[36] Shawahna R, Uchida Y, Declèves X, Ohtsuki S, Yousif S, Dauchy S, et al Transcriptomic and quantitative proteomic analysis of transporters and drug metabolizing enzymes in freshly isolated human brain microvessels. Mol Pharm. American Chemical Society; 2011;8: 1332–1341. 10.1021/mp200129p 21707071

[37] Do TM, Noel-Hudson M-S, Ribes S, Besengez C, Smirnova M, Cisternino S, et al ABCG2- and ABCG4-mediated efflux of amyloid-β peptide 1–40 at the mouse blood-brain barrier. J Alzheimers Dis. IOS Press; 2012;30: 155–166. 10.3233/JAD-2012-112189 22391220

[38] Bauer M, Karch R, Zeitlinger M, Stanek J, Philippe C, Wadsak W, et al Interaction of 11C-tariquidar and 11C-elacridar with P-glycoprotein and breast cancer resistance protein at the human blood-brain barrier. J Nucl Med. Society of Nuclear Medicine; 2013;54: 1181–1187. 10.2967/jnumed.112.118232 23833270 PMC3882137

[39] Sapunar D, Kostic S, Banozic A, Puljak L. Dorsal root ganglion—a potential new therapeutic target for neuropathic pain. J Pain Res. Dove Press; 2012;5: 31–38. 10.2147/JPR.S26603 22375099 PMC3287412

[40] Yao X, Chang AY, Boulpaep EL, Segal AS, Desir GV. Molecular cloning of a glibenclamide-sensitive, voltage-gated potassium channel expressed in rabbit kidney. J Clin Invest. American Society for Clinical Investigation; 1996;97: 2525–2533. 10.1172/JCI118700 8647945 PMC507338

[41] Myngheer N, Allegaert K, Hattersley A, McDonald T, Kramer H, Ashcroft FM, et al Fetal macrosomia and neonatal hyperinsulinemic hypoglycemia associated with transplacental transfer of sulfonylurea in a mother with KCNJ11-related neonatal diabetes. Diabetes Care. American Diabetes Association; 2014;37: 3333–3335. 10.2337/dc14-1247 25231897 PMC5894804

[42] Fendler W, Pietrzak I, Brereton MF, Lahmann C, Gadzicki M, Bienkiewicz M, et al Switching to sulphonylureas in children with iDEND syndrome caused by KCNJ11 mutations results in improved cerebellar perfusion. Diabetes Care. American Diabetes Association; 2013;36: 2311–2316. 10.2337/dc12-2166 23462667 PMC3714477

[43] Mourre C, Widmann C, Lazdunski M. Sulfonylurea binding sites associated with ATP-regulated K+ channels in the central nervous system: autoradiographic analysis of their distribution and ontogenesis, and of their localization in mutant mice cerebellum. Brain Res. 1990;519: 29–43. 2118822 10.1016/0006-8993(90)90057-i

[44] Karschin C, Ecke C, Ashcroft FM, Karschin A. Overlapping distribution of K(ATP) channel-forming Kir6.2 subunit and the sulfonylurea receptor SUR1 in rodent brain. FEBS Lett. 1997;401: 59–64. 9003806 10.1016/s0014-5793(96)01438-x

