# Correction: Systemic Administration of Glibenclamide Fails to Achieve Therapeutic Levels in the Brain and Cerebrospinal Fluid of Rodents

**DOI:** 10.1371/journal.pone.0215989

**Published:** 2019-04-18

**Authors:** Carolina Lahmann, Holger B. Kramer, Frances M. Ashcroft

The following information is missing from the Funding statement: We thank the European Research Council for funding (grant no. 322620 to FMA).
