# Supplementary figures and images for: Systemic Administration of Glibenclamide Fails to Achieve Therapeutic Levels in the Brain and Cerebrospinal Fluid of Rodents
Source: PLoS One. 2015 Jul 30;10(7):e0134476. doi: 10.1371/journal.pone.0134476 (PMC4520580; doi:10.1371/journal.pone.0134476)

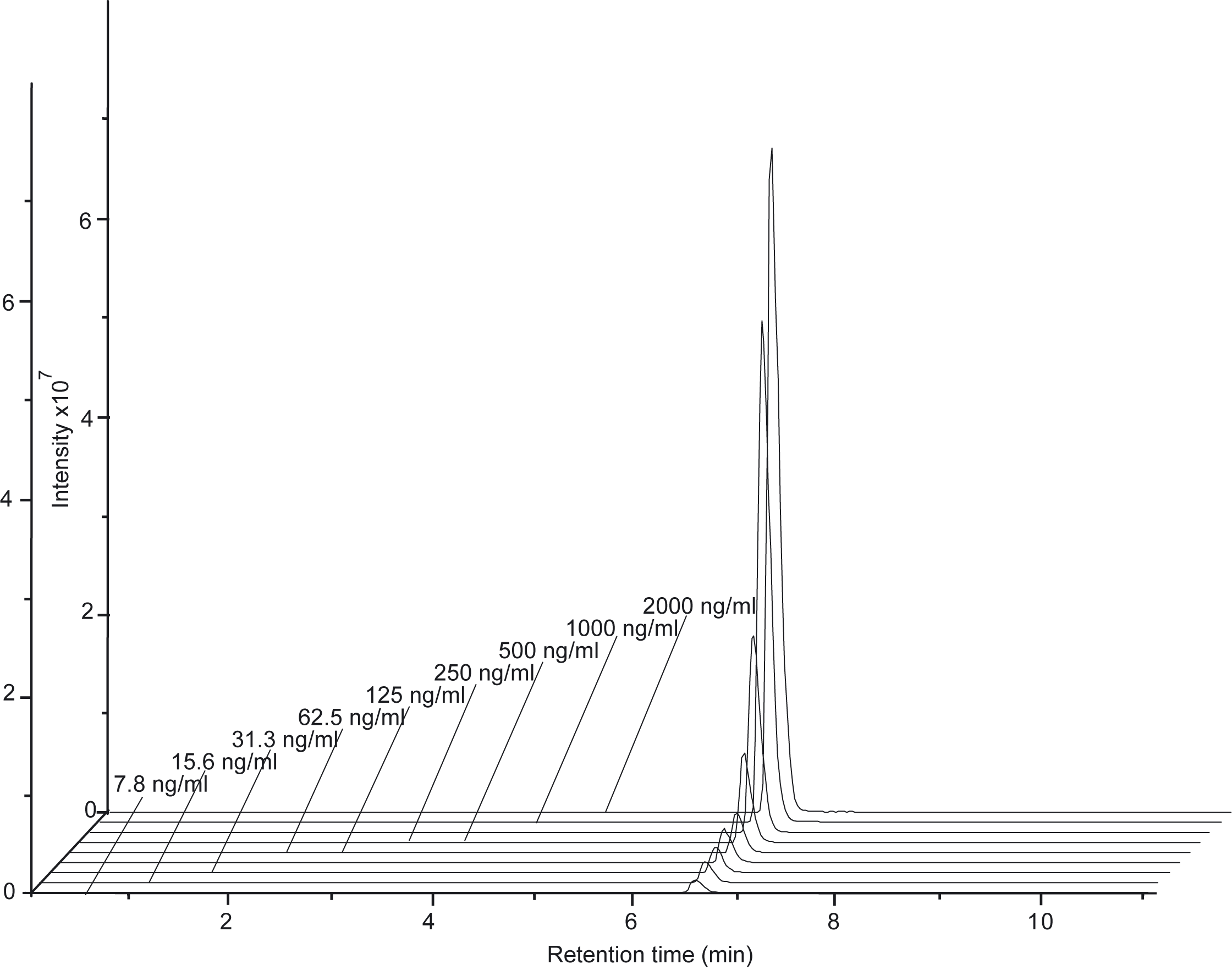

Supplement: S1 Fig — LC-MS chromatograms displaying the intensity of transition m/z 494.1→369.0 (6.6min retention time) for spiked plasma samples with the following concentrations: 7.8, 15.6, 31.3, 62.5, 125, 250, 500, 1000 and 2000ng/ml. (TIF) [file pone.0134476.s002.tif]

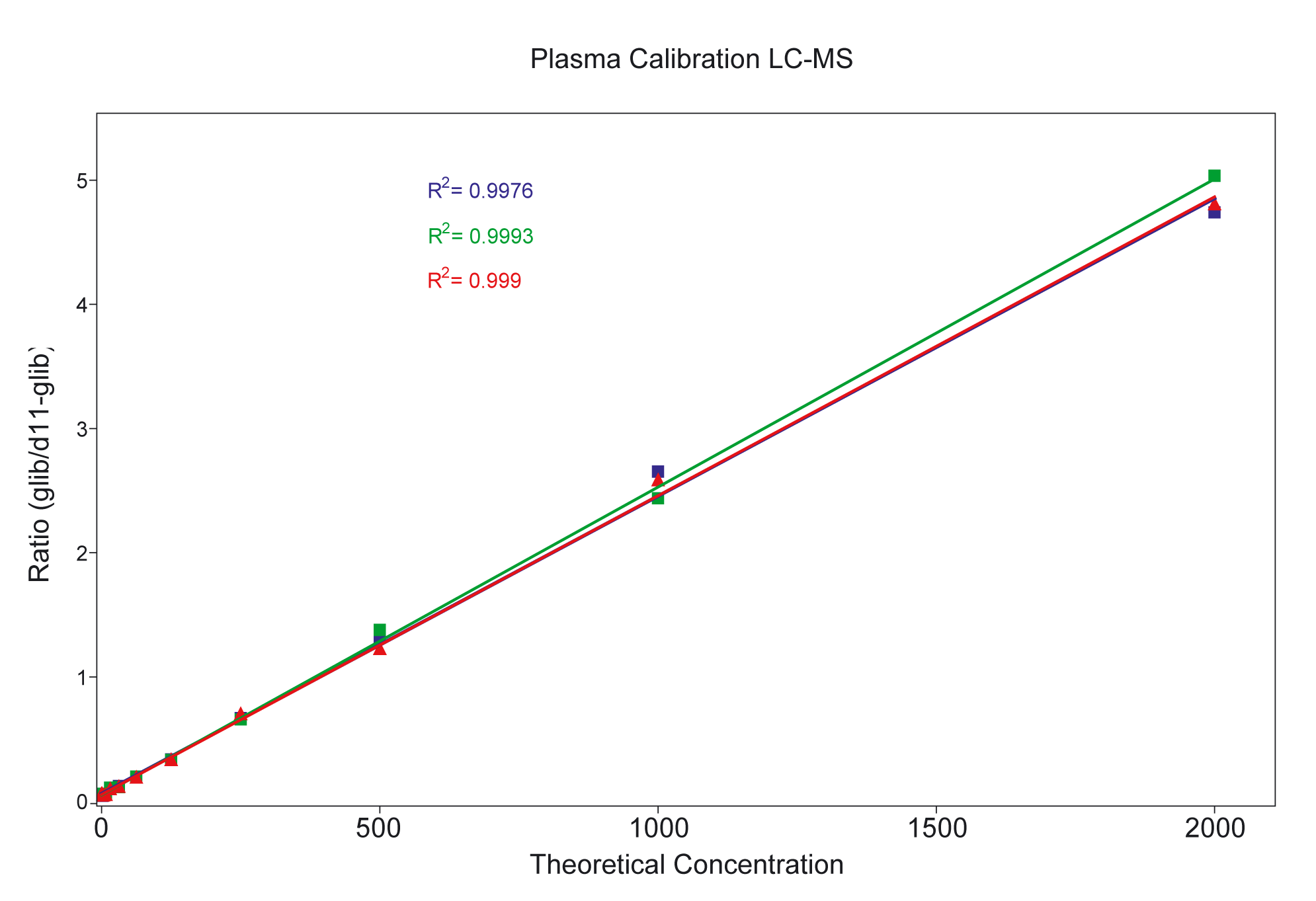

Supplement: S2 Fig — Calibration curves obtained from triplicate analysis of spiked plasma standards by LC-MS (SRM acquisition) immediately prior to injection of unknown samples. Replicates for each set of replicates and the corresponding R2 values are displayed in dark blue, green and red. (TIF) [file pone.0134476.s003.tif]

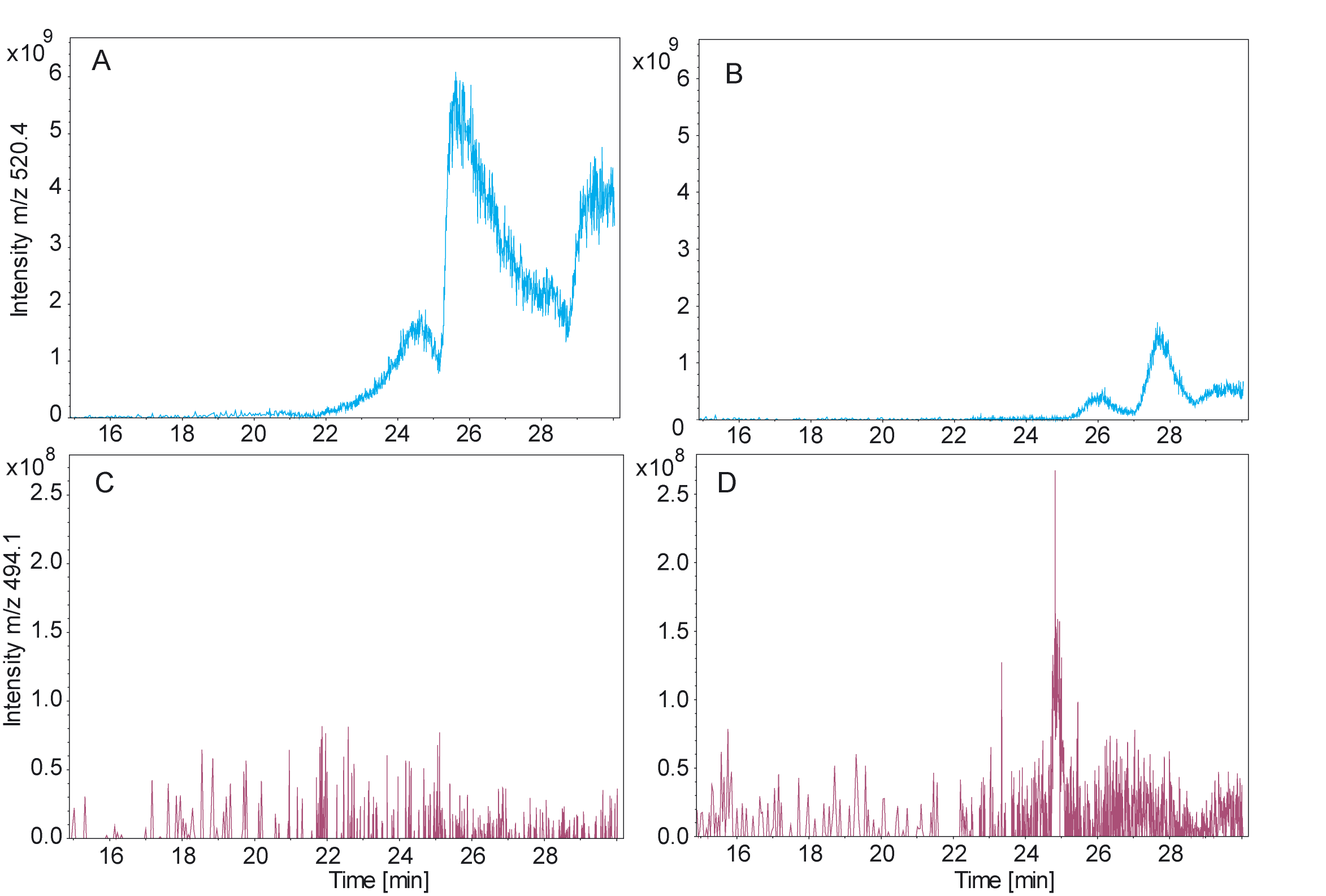

Supplement: S3 Fig — Analysis of plasma extracts by nanoflow LC-MS showing extracted ion chromatograms of m/z 520.4 phospholipid contaminant (cyan) and m/z 494.1 glibenclamide (magenta).(A,C) protein precipitation and filtration (note different scales in A and C). (B,D) reverse phase C18-SPE (note different scales in B and D). (TIF) [file pone.0134476.s004.tif]
