# Supplementary material for: Systemic Administration of Glibenclamide Fails to Achieve Therapeutic Levels in the Brain and Cerebrospinal Fluid of Rodents
Source: PLoS One. 2015 Jul 30;10(7):e0134476. doi: 10.1371/journal.pone.0134476 (PMC4520580; doi:10.1371/journal.pone.0134476)
Supplement: S1 Table — Calibration curves obtained from triplicate analysis of spiked plasma standards by LCMS (SRM acquisition) on three separate days. Linear regression was carried out by the least squares method with a 1/y2 weighting. Precision was expressed as percentage coefficient of variation (%CV) and was calculated as standarddeviationmeanconcentration×100. (DOCX) [file pone.0134476.s005.docx]

| **y=bx+a** |  |  |  |  |  |  |  |
| --- | --- | --- | --- | --- | --- | --- | --- |
|  | **Day 1** | **Day 2** | **Day 3** | **Mean** | **St. Dev.** |  |  |
| **Slope [b]** | 1.0833 | 1.0715 | 1.1123 | 1.0891 | 0.0210 |  |  |
| **Intercept [a]** | 0.0330 | 0.0356 | 0.0307 | 0.0331 | 0.0024 |  |  |
| **Correlation [r]** | 0.9963 | 0.9980 | 0.9976 | 0.9973 | 0.0009 |  |  |
|  | | | | | | | |
| **Concentration [ng/ml]** | **Day 1** | **Day 2** | **Day 3** | **Mean conc. (ng/ml)** | **St. Dev.** | **Precision CV (%)** | **Accuracy (%)** |
| 5 | 4.21 | 3.79 | 5.19 | 4.40 | 0.72 | 16.27 | 87.94 |
| 10 | 11.73 | 10.39 | 11.92 | 11.35 | 0.84 | 7.37 | 113.46 |
| 50 | 55.58 | 56.20 | 53.27 | 55.02 | 1.55 | 2.81 | 110.03 |
| 200 | 224.04 | 229.68 | 230.57 | 228.10 | 3.54 | 1.55 | 114.05 |
| 500 | 529.25 | 520.70 | 517.11 | 522.35 | 6.23 | 1.19 | 104.47 |
| 1000 | 977.78 | 974.71 | 984.40 | 978.96 | 4.95 | 0.51 | 97.90 |
| 2000 | 1781.66 | 1739.07 | 1781.83 | 1767.52 | 24.63 | 1.39 | 88.38 |
| 3000 | 3052.58 | 3034.49 | 2901.66 | 2996.24 | 82.41 | 2.75 | 99.87 |
| 4000 | 3728.87 | 3986.58 | 3850.91 | 3855.45 | 128.91 | 3.34 | 96.39 |
