# Supplementary material for: Systemic Administration of Glibenclamide Fails to Achieve Therapeutic Levels in the Brain and Cerebrospinal Fluid of Rodents
Source: PLoS One. 2015 Jul 30;10(7):e0134476. doi: 10.1371/journal.pone.0134476 (PMC4520580; doi:10.1371/journal.pone.0134476)
Supplement: S2 Table — Mean accuracy, relative standard deviation (SD) and coefficient of variation (CV) for intra-day (n = 5) and inter-day measurements (n = 15). Accuracy is defined as the percentage of the theoretical concentration. Relative SD is defined as standarddeviationtheoreticalconcentration×100. Precision is expressed as the percentage coefficient of variation (%CV) and calculated as standarddeviationmeanconcentration×100. (DOCX) [file pone.0134476.s006.docx]

| **Glibenclamide concentration [ng/ml]** |  | **Day 1** | **Day 2** | **Day 3** | **Inter-day (n=15)** |
| --- | --- | --- | --- | --- | --- |
| 40 (n=5) | Mean accuracy (%) | 92.55 | 85.67 | 83.82 | 87.35 |
|  | Relative SD (%) | 8.57 | 5.02 | 5.55 | 7.22 |
|  | CV (%) | 9.26 | 5.86 | 6.62 | 8.27 |
| 350 (n=5) | Mean accuracy (%) | 102.45 | 101.88 | 102.29 | 102.21 |
|  | Relative SD (%) | 4.92 | 2.43 | 2.61 | 3.26 |
|  | CV (%) | 4.80 | 2.39 | 2.55 | 3.19 |
| 3500 (n=5) | Mean accuracy (%) | 100.75 | 96.25 | 100.88 | 99.53 |
|  | Relative SD (%) | 3.58 | 5.12 | 4.12 | 4.42 |
|  | CV (%) | 3.55 | 5.28 | 4.09 | 4.44 |
